# Altered resting-state functional connectivity of raphe nucleus is associated with tremor in Parkinson’s disease

**DOI:** 10.3389/fnagi.2025.1709735

**Published:** 2025-12-31

**Authors:** Qianyi Zheng, Dongling Zhang, Junyan Sun, Junling Wang, Lili Chen, Xuemei Wang, Tao Wu

**Affiliations:** 1 Department of Neurology, Center for Movement Disorders, Beijing Tiantan Hospital, Capital Medical University, Beijing, China; 2 Department of Neurology, Tianjin Huanhu Hospital, Tianjin, China

**Keywords:** functional connectivity, Parkinson’s disease, raphe nuclei, serotonergic system, tremor

## Abstract

**Introduction:**

Tremor is a prevalent and disabling motor symptom in Parkinson’s disease (PD). The role of the serotonergic system in Parkinsonian tremor remains unclear. We aimed to investigate whether functional connectivity (FC) of the dorsal (DRN) and median (MRN) raphe nuclei is associated with tremor in PD.

**Methods:**

Forty PD patients with tremor dominant (TD-PD), 42 PD patients with postural instability and gait disturbance dominant (PIGD-PD), and 40 healthy controls (HCs) were enrolled. Resting-state functional MRI was used to investigate altered FC of the DRN and MRN in TD-PD patients compared to HCs and PIGD-PD patients. Subsequently, correlations between FC of the raphe nuclei and motor-related clinical variables were analyzed.

**Results:**

Both TD-PD and PIGD-PD patients showed reduced FC of the raphe nuclei compared to HCs. TD-PD patients demonstrated a more pronounced reduction in FC for both DRN and MRN across extensive brain regions, such as the sensorimotor cortex, temporal cortex, occipital cortex, and cerebellum, relative to PIGD-PD patients. Correlation analysis revealed that FC of both DRN and MRN was negatively correlated with tremor severity, including the total tremor score, rest tremor scores (amplitude, constancy, and index of severity), and postural tremor score. Our findings indicate significant hypoconnectivity of both DRN and MRN in TD-PD patients. Moreover, both DRN and MRN related functional networks exhibited correlations with tremor severity.

**Discussion:**

These results support the association between serotonergic dysfunction and Parkinsonian tremor, suggesting that both DRN and MRN may play critical roles in the pathogenesis of tremor in PD.

## 1 Introduction

Parkinson’s disease (PD) is the second most common neurodegenerative disorder, characterized pathologically by the degeneration of dopaminergic neurons in the substantia nigra pars compacta (Braak et al., 2003; Samii et al., 2004). Clinically, PD manifests with complex and heterogeneous motor symptoms, including tremor, bradykinesia, postural instability, and rigidity. Based on the predominant motor symptoms, PD patients can be categorized into tremor-dominant (TD) and postural instability/gait difficulty (PIGD) subtypes (Thenganatt and Jankovic, 2014), which exhibit variable clinicopathologic phenotypes and therapeutic responses. Among these cardinal features, tremor in PD is particularly complex and poorly understood, with variable responsiveness to dopaminergic therapy (Fishman, 2008). Consequently, a deeper understanding of the pathophysiological mechanisms underlying Parkinsonian tremor is urgently required. Previous studies have suggested that Parkinsonian tremors arise from dysfunction of both basal ganglia and cerebello-thalamo-cortical (CTC) circuits (Helmich et al., 2012). Diminished projections from the serotonergic (5-hydroxytryptamine, 5-HT) system may contribute to the impairments in these circuits (Helmich and Dirkx, 2017). However, our knowledge regarding the association between the serotonergic system and Parkinsonian tremor remains limited.

Serotonin neurons mainly originate from the dorsal (DRN) and median (MRN) raphe nuclei. DRN and MRN provide parallel and overlapping projections to various brain regions, such as the cerebral cortex, basal ganglia, limbic system, and cerebellum (Pollak Dorocic et al., 2014; Commons, 2016). Specifically, the DRN predominantly projects to the cerebral cortex and basal ganglia, whereas the MRN preferentially projects to the prefrontal cortex, cingulate cortex, and hippocampus (Commons, 2016). Molecular imaging studies indicate that more severe serotonin dysfunction correlates with more pronounced Parkinsonian tremor (Loane et al., 2013; Qamhawi et al., 2015; Pasquini et al., 2018). However, these studies have only assessed regional serotonergic function, the association between the raphe nuclei-related networks and Parkinsonian tremor remains unclear. To date, only a few studies have investigated functional connectivity (FC) alterations of the raphe nuclei in PD patients. These studies demonstrated reduced FC of the raphe nuclei in PD compared to healthy controls (HCs), with the reduced connectivity linked to non-motor symptoms (Shen et al., 2022; Wang et al., 2022) and freezing of gait (Lv et al., 2022). However, the association between the raphe nuclei-related functional networks and Parkinsonian tremor has not been investigated.

Therefore, in this study, we utilized resting-state functional MRI (rs-fMRI) to examine the differential FC of the DRN and MRN in TD-PD compared to HC and PIGD-PD groups. Additionally, we conducted correlation analyses between the FC of the raphe nuclei and clinical characteristics (including tremor, PIGD, and rigidity scores). Our findings will enhance the understanding of the role of the serotonergic system in Parkinsonian tremor, and clarify whether both DRN and MRN are involved in tremor in PD.

## 2 Materials and methods

### 2.1 Participants

A total of 150 participants were initially enrolled in this study and provided written informed consent. Among them, 9 PD patients and 6 HCs were excluded due to excessive head movements. Additionally, 13 PD patients classified as indeterminate PD were also excluded from the analysis. Therefore, 82 PD (40 TD-PD and 42 PIGD-PD) and 40 HCs were included in the final analysis from two independent cohorts. Specifically, cohort 1 consisted of 96 participants (TD-PD: 31, PIGD-PD: 35, HCs: 30), recruited between June 2017 and May 2021 at the Department of Neurology, Xuanwu Hospital of Capital Medical University. Cohort 2 included 26 participants (TD-PD: 9, PIGD-PD: 7, HC: 10), recruited between March 2021 and October 2021 at the Department of Neurology, Beijing TianTan Hospital of Capital Medical University. The diagnosis of PD was based on the Movement Disorder Society (MDS) Clinical Diagnostic Criteria (Postuma et al., 2015). HCs were enrolled from the community and met the following criteria: no history of neurological or psychiatric disorders, no family history of movement disorders, no cognitive or behavioral abnormalities, no evident cerebral lesions on MRI images, and no contraindications for MRI scanning.

### 2.2 Clinical evaluations

In addition to demographic information, all participants underwent clinical screening for motor and nonmotor symptoms, including Hamilton Depression Scale (HAMD) (Hamilton, 1960), Montreal Cognitive Assessment (MoCA) (Nasreddine et al., 2005), Pittsburgh Sleep Quality Index (PSQI) (Mollayeva et al., 2016), Epworth Sleepiness Scale (ESS) (Boyes et al., 2017), and Rapid Eye Movement Sleep Behavior Disorder Questionnaire (Hong Kong version) (RBD-HK) (Li et al., 2010). The MDS Unified Parkinson’s Disease Rating Scale (MDS-UPDRS) (Goetz et al., 2007) and Hoehn and Yahr (H&Y) disability scale were assessed in PD patients while off their medication for at least 12 h. Disease duration and levodopa equivalent daily dosage (LEDD) (Tomlinson et al., 2010) were also obtained from all PD patients. In addition, all PD patients underwent (123) I-ioflupane SPECT scans to assess the striatal specific binding ratio (SBR). Images were iteratively reconstructed with attenuation and scatter correction. All scans were then analyzed centrally using DaTQUANT software (v2.0, GE Healthcare), which applies standardized volume-of-interest (VOI) templates. The SBR is defined as the mean counts of the striatal VOI (background-subtracted) divided by the mean counts of the occipital lobe VOI (Iwabuchi et al., 2021).

### 2.3 Subtype classification and clinical feature evaluation

PD patients were classified into TD-PD, PIGD-PD, and indeterminate subtypes based on the MDS-UPDRS parts II and III (Stebbins et al., 2013). Specifically, the ratio of the mean MDS-UPDRS tremor scores (items 2.10, 3.15a, 3.15b, 3.16a, 3.16b, 3.17a, 3.17b, 3.17c, 3.17d, 3.17e, and 3.18) to the mean MDS-UPDRS PIGD scores (items 2.12, 2.13, 3.10, 3.11, and 3.12) was calculated for each patient. Patients with ratios ≥1.15 were categorized as TD-PD, those with ratios ≤ 0.90 were categorized as PIGD-PD, and those with ratios between 0.90 and 1.15 were categorized as indeterminate subtypes. Patients in the indeterminate subtype were excluded from this study.

For each patient, we calculated tremor-related scores [total tremor score (total score of items 2.10, 3.15, 3.16, 3.17, and 3.18), rest tremor amplitude (highest and total score of item 3.17), rest tremor constancy (item 3.18), postural tremor of the hands (item 3.15) and kinetic tremor of the hands (item 3.16)], PIGD score (total score of items 2.12, 2.13, 3.10, 3.11 and 3.12), rigidity score (total score of item 3.3), body bradykinesia score (item 3.14) and bradykinesia score (items 3.4, 3.5, 3.6, 3.7 and 3.8) (Qamhawi et al., 2015; Pasquini et al., 2018). The index of rest tremor severity was also calculated by multiplying the total amplitude score and constancy score.

### 2.4 MRI data acquisition

Each patient was instructed to lie still with eyes closed, awake, head position steady, and without thinking of anything during the rs-fMRI scanning. All patients were scanned in the off-medication state (after withdrawing their medication for at least 12 h). MRI data were acquired using a 3T Magnetom Skyra scanner for cohort 1, and a 3.0T GE SIGNA Premier scanner for cohort 2. Rs-fMRI images in cohort 1 were obtained using a single-shot spin-echo echoplanar imaging (SE-EPI) sequence with the following parameters: repetition time (TR) = 2000 ms, echo time (TE) = 30 ms, field of view (FOV) = 220 × 220 mm^2^, image matrix size = 64 × 64, flip angle = 90°, voxel size = 3.4 × 3.4 × 3 mm^3^, 35 axial slices with no gap, 176 repetitions, scanning time = 5 min 52 s. Rs-fMRI data in cohort 2 were obtained with the following parameters: TR = 1,000 ms, TE = 39 ms, FOV = 208 × 208 mm^2^, image matrix size = 86 × 86, flip angle = 64°; voxel size = 2.4 × 2.4 × 2.4 mm^3^, 65 axial slices with no gap, 330 repetitions, scanning time = 5 min 30 s. T1-weighted anatomic images were scanned using a magnetization-prepared 3D rapid gradient echo (MPRAGE) sequence in both cohorts. The imaging parameters in cohort 1 were as follows: TR = 2,530 ms, TE = 2.98 ms, FOV = 224 × 256 mm^2^, flip angle = 7°, voxel size 1 × 1 × 1 mm^3^; 192 slices with no gap. The parameters in cohort 2 were as follows: TR = 1952 ms, TE = 2.20 ms, FOV = 256 × 256 mm^2^, flip angle = 8°, voxel size 0.5 × 0.5 × 0.5 mm^3^, 376 slices with no gap.

### 2.5 MRI data preprocessing

After converting to the NIFTI format, imaging data were preprocessed using FSL.^1^ Briefly, the main steps included removing the first 10 time points, motion correcting using MCFILRT [motion correction tool based FMRIB’s Linear Registration Tool (FLIRT)], spatial normalizations to the Montreal Neurological Institute (MNI) template using 12-parameter affine transformation, and registration of fMRI images to MNI space via high-resolution T1 images using FSL FLIRT and FNIRT (FMRIB’s Nonlinear Image Registration Tool) and resampling into 3-mm isotropic voxels. Furthermore, as located in the brainstem, fMRI signals in the raphe nuclei are susceptible to physiological noises, such as respiratory and cardiac noise. Therefore, independent component analysis (ICA) was performed to denoise the fMRI signal, by using MELODIC (Multivariate Exploratory Linear Optimized Decomposition into Independent Components) in FSL, which could decompose a single-subject’s fMRI data into different spatial (maps) and temporal (time series) components. Then, the signal and noise of independent components were manually identified, and noises were labeled and removed (Griffanti et al., 2016). The examples of the signals and noises are shown in Supplementary Table S1, Figure S1. Then, spatial smoothing with a 3-mm full width at half maximum Gaussian smoothing kernel, detrend, and temporal high-pass filtering above 0.01–0.08 Hz were performed using DAPBI (version 8.2; https://rfmri.org/DPABI). Participants were excluded if they had >3 mm of framewise displacement, or >3 mm or 3° of translational or rotational movements.

### 2.6 Definition of raphe nuclei and functional connectivity analysis

The DRN and MRN were chosen as regions of interest (ROIs) for whole-brain FC analysis (Supplementary Figure S2). Spherical ROIs with a 4-mm radius were employed as they provide sufficient voxels to ensure signal stability and mitigate partial volume effects, whereas a smaller ROI would increase noise susceptibility and a larger one would reduce spatial specificity by incorporating non-target signals. The centers were located at *x* = 2, *y* = −26, *z* = −20 for DRN, and *x* = 0, *y* = −31, *z* = −24 for MRN (MNI space) based on previous studies (Kranz et al., 2012; Beliveau et al., 2015; Faulkner et al., 2018). For each ROI, a voxel-wise FC analysis was performed by computing Pearson’s correlation coefficients between the mean time series of all voxels within the ROI and the time series of each voxel within the gray matter. The individual correlation coefficients were transformed to *z*-values by using Fisher’s *r*-to-*z* transformation to improve the normality. Since our data were collected across two cohorts, to control the multi-center site effect, ComBat Harmonization was applied to correct for scanner/site effects on the FC maps by using the Harmonization module of DPABI.

### 2.7 Statistical analysis

Demographic and clinical features were analyzed using IBM SPSS Statistics 27 and GraphPad Prism 9.0. The Kolmogorov–Smirnov test was used to assess the normal distribution of the data. A one-way analysis of variance (ANOVA) (for age), Kruskal-Wallis test (for years of education, HAMD, MoCA, RBD-HK, and RSQI scores), and Chi-square test (for gender) were used to examine differences among the three groups. Mann–Whitney U tests (for H&Y, LEDD, duration, MDS-UPDRS III, total tremor score, and PIGD score) were used to evaluate differences between the TD-PD and PIGD-PD groups. All tests were two-tailed, and a *p*-value <0.05 was considered statistically significant.

The voxel-wise analysis was conducted using SPM12.^2^ One-sample *t*-tests were conducted to assess the connectivity profile of each ROI, and then the FC maps were generated for all three groups using BrainNet Viewer.^3^ Differences in FC among the three groups were analyzed using analysis of covariance (ANCOVA) with age, gender, years of education, and head movement as covariates, and *post hoc* tests were used for between-group comparisons. The significant level was set at false discovery rate (FDR)-corrected *p* < 0.05 at the voxel level with a cluster size ≥10 voxels.

Based on the data distribution, we used Spearman partial correlation analyses to evaluate the relationship between FC values and clinical variables in pooled PD patients (including all TD-PD and PIGD-PD patients) and the subgroups. The FC values were extracted from the significantly different clusters identified from the contrast comparing TD-PD and PIGD-PD subtypes. Subsequently, correlation analyses with age, sex, education, head motion, SBR, and ESS as covariates were conducted between the averaged FC values of each cluster and motor-related clinical variables, including tremor-related scores [total tremor score, rest tremor scores (total and highest amplitude, constancy, and index of severity), postural and kinetic tremor scores], PIGD score, rigidity score, body bradykinetic score, and bradykinetic score. In addition, depending on data distribution, Pearson or Spearman correlation analysis was selected to evaluate the association between FC values and SBR and non-motor clinical variables, including the scores of HAMD, MoCA, ESS, PSQI, and RBDQ-HK. A Bonferroni-corrected *p*-value <0.05 was considered statistically significant.

## 3 Results

### 3.1 Characteristics of demographic and clinical features

The demographic and clinical features of all participants are summarized in Table 1. No significant differences were observed among the three groups in age, gender, years of education, MoCA, or PSQI score. Both TD-PD and PIGD-PD patients had higher HAMD and RBDQ-HK scores compared to HCs. Compared to PIGD-PD patients, TD-PD patients exhibited a higher total tremor score and a lower PIGD score. No significant differences were observed in disease duration, H&Y stage, LEDD, MDS-UPDRS III score, and non-motor scores between TD-PD and PIGD-PD patients.

**Table 1:** Demographic and clinical characteristics of participants.

Characteristics	HC (*n* = 40)	TD-PD (*n* = 40)	PIGD-PD (*n* = 42)	*p*-value
Age (year)	61.75 ± 8.26	61.83 ± 7.07	61.36 ± 9.07	0.9619^a^
Gender (male, %)	13, 32.50%	23, 57.50%	22, 52.38%	0.0604^b^
Education (year)	10.16 ± 2.86	9.85 ± 3.89	11.24 ± 3.48	0.1347^c^
Duration (month)	–	54.35 ± 50.76	53.71 ± 38.39	0.5032^d^
H&Y	–	1.83 ± 0.55	2.08 ± 0.77	0.0929^d^
LEDD	–	518.66 ± 357.46	517.34 ± 396.82	0.9897^e^
SBR	–	2.31 ± 0.42	2.21 ± 0.40	0.2803^d^
MDS UPDRS III	–	25.65 ± 11.36	28.21 ± 13.94	0.3653^e^
Total tremor score	–	8.93 ± 3.74	3.10 ± 3.11	**<0.0001**^**d**^
PIGD score	–	1.60 ± 1.08	4.83 ± 3.27	**<0.0001**^**d**^
HAMD	2.74 ± 3.10	5.70 ± 4.35	6.93 ± 5.42	**0.0006**^**c**^
MoCA	24.68 ± 3.20	23.35 ± 3.72	23.79 ± 4.00	0.3111^c^
ESS	3.75 ± 2.62	4.08 ± 3.92	5.03 ± 5.72	0.9394^c^
PSQI	5.93 ± 4.51	7.41 ± 4.78	6.65 ± 3.71	0.3642^c^
RBDQ-HK	8.57 ± 6.23	17.81 ± 16.66	19.24 ± 16.68	**0.0383**^**c**^

ESS, Epworth Sleepiness Scale; HAMD, Hamilton Depression Scale; HC, healthy controls; H&Y, Hoehn and Yahr disability scale; LEDD, levodopa-equivalent daily dose; MDS-UPDRS, MDS Unified Parkinson’s Disease Rating Scale; MoCA, Montreal Cognitive Assessment; PD, Parkinson’s disease; PIGD, postural instability/gait difficulty; PSQI, Pittsburgh Sleep Quality Index; RBD-HK, Rapid eye movement sleep Behavior Disorder Questionnaire (Hong Kong version); SBR, striatal binding ratios; TD, tremor-dominant. Test stats: a, ANOVA; b, Chi-square; c, Kruskal-Wallis test; d, Mann–Whitney U tests; e, *T*-test. Values are presented as mean ± standard deviation (SD). Bold values showed significant differences (*p*-value <0.05).

### 3.2 Functional connectivity of raphe nuclei

The within-group functional connectivity analysis revealed extensive common and distinct positive connectivity between DRN and MRN. In addition, significant differences in FC of the raphe nuclei were observed across extensive brain regions among TD-PD, PIGD-PD, and HCs (*p* < 0.05, FDR corrected; Supplementary Figures S3, S4).

#### 3.2.1 Differences in FC between TD-PD and HC

**Figure 1:**
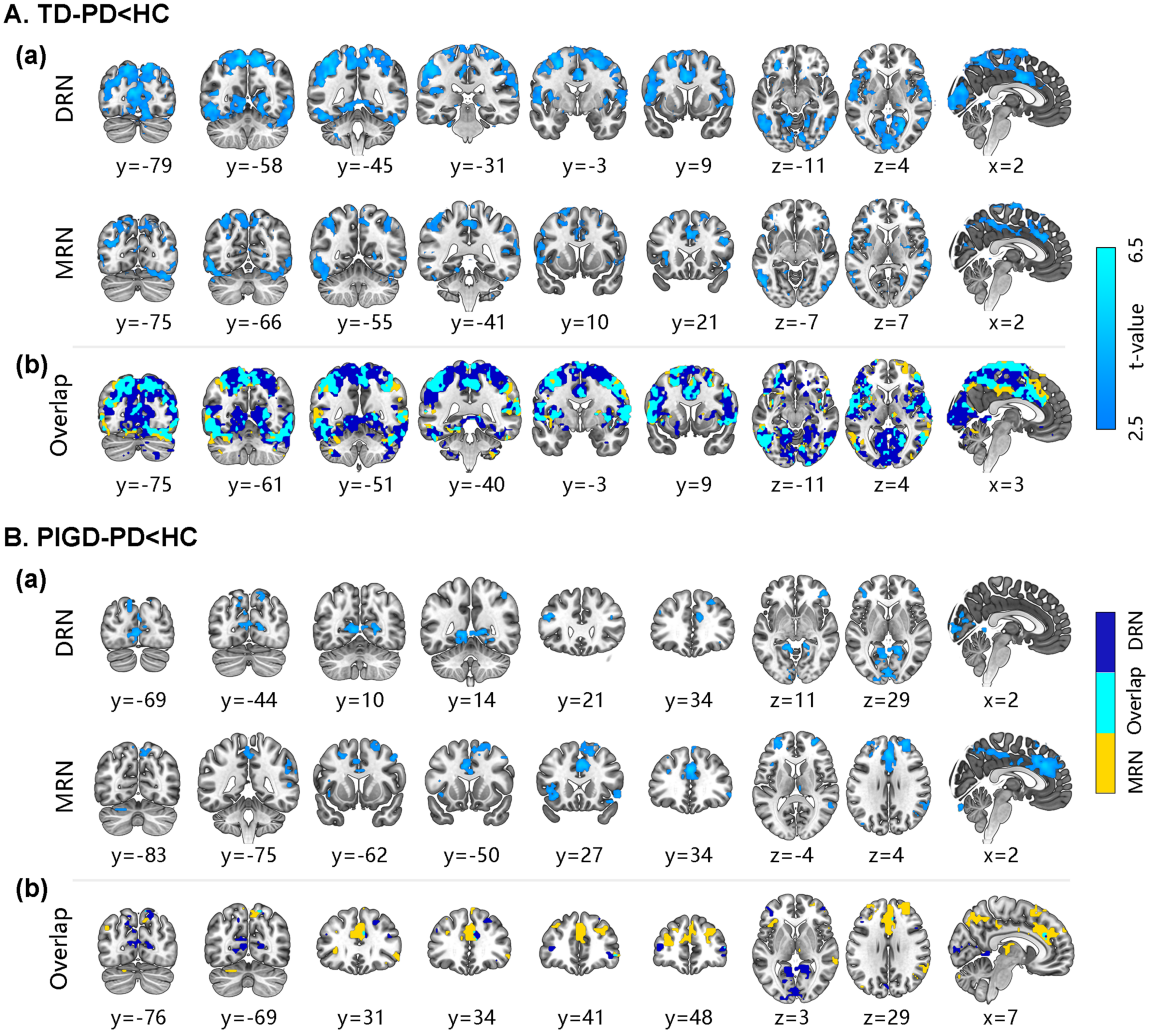
Alteration of functional connectivity for the raphe nuclei in PD subgroups. The brain regions of decreased connectivity with DRN and MRN in TD-PD **(A)** and PIGD-PD patients **(B)** compared with HCs. **(Aa, Ba)** Significant group differences in FC values for the DRN and MRN (*p*-value <0.05, FDR-corrected, MNI152 space, cluster size ≥10 voxels). **(Ab, Bb)** Similarities and differences in spatial maps of FC between DRN and MRN. The brain areas with orange are unique to DRN, those with blue are specific to MRN, and those with cyan are common to both. DRN, dorsal raphe nucleus; MRN, median raphe nucleus; HC, healthy control; PIGD-PD, postural instability, and gait difficulty-dominant Parkinson’s disease; TD-PD, Tremor-dominant Parkinson’s disease.

Compared to HCs, both the DRN and MRN exhibited hypoconnectivity with the bilateral sensorimotor cortex, right temporal cortex, left occipital cortex, bilateral median cingulate and paracingulate gyri (DCG), left putamen and pallidum, right thalamus, and bilateral posterior cerebellum in TD-PD. In addition, the DRN had hypoconnectivity with the bilateral parietal cortex, right occipital cortex, bilateral limbic system, and left caudate and thalamus, while the MRN had hypoconnectivity with the right frontal cortex, bilateral temporal cortex, right occipital cortex, right putamen, and bilateral posterior cerebellum (*p* < 0.05, FDR corrected; Figure 1A).

#### 3.2.2 Differences in FC between PIGD-PD and HC

Compared to HCs, both the DRN and MRN exhibited reduced FC with the bilateral frontal cortex, right parietal cortex, and left posterior cerebellum in PIGD-PD patients. Furthermore, the DRN showed reduced connectivity with the bilateral superior parietal gyrus and occipital cortex, while the MRN had reduced connectivity with the bilateral sensorimotor cortex, temporal cortex, middle occipital gyrus, limbic system, thalamus, and right posterior cerebellum (p < 0.05, FDR corrected; Figure 1B).

#### 3.2.3 Differences in FC between TD-PD and PIGD-PD

**Figure 2:**
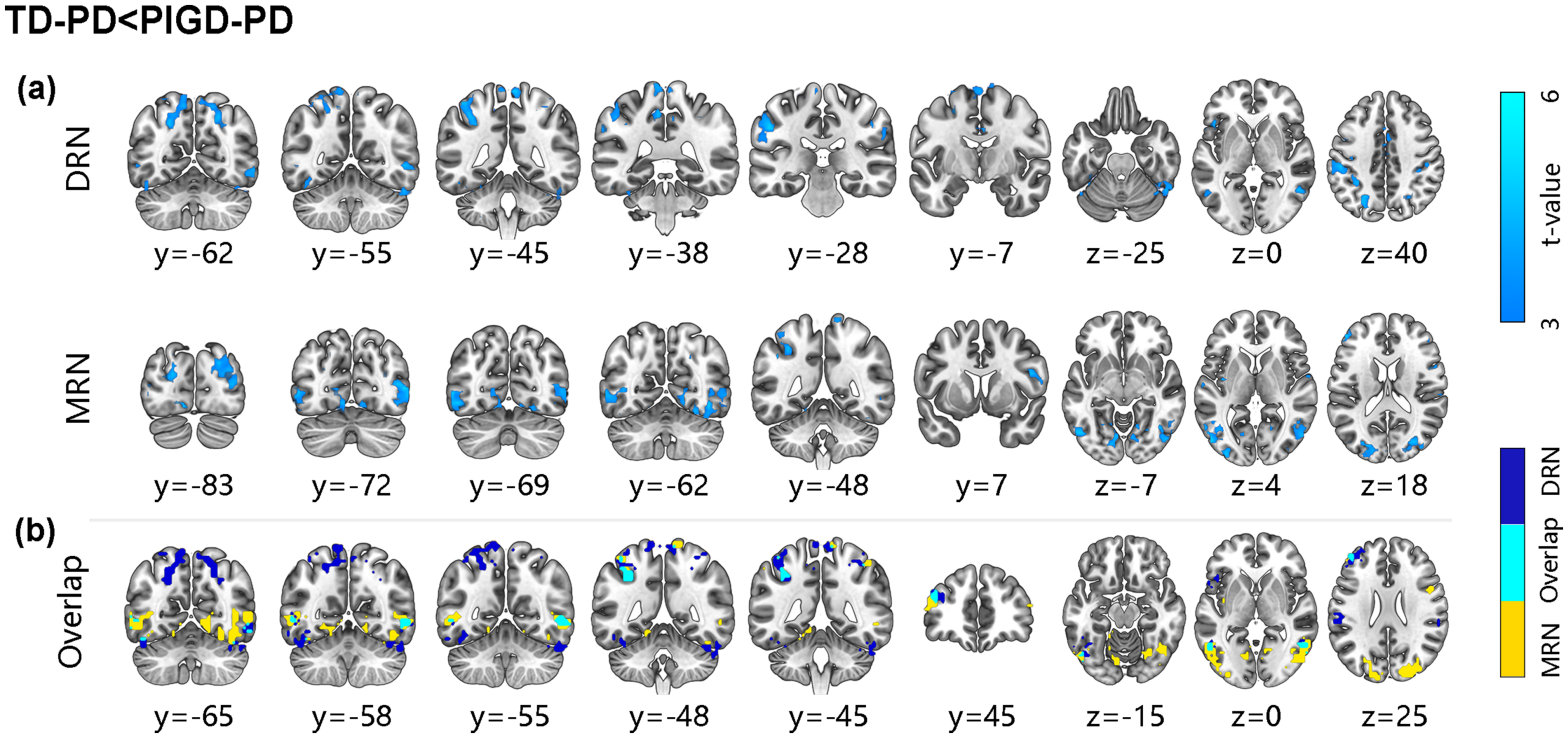
Alteration of functional connectivity for the raphe nuclei between patients with TD-PD and PIGD-PD. **(a)** Decreased functional connectivity of DRN and MRN in TD-PD compared to PIGD-PD (*p*-value <0.05, FDR-corrected, MNI152 space, cluster size ≥10 voxels). **(b)** Common and unique reduced functional connectivity of DRN and MRN in TD-PD than PIGD-PD. The brain areas with blue are unique to DRN, those with yellow are specific to MRN, and those with cyan are common to both.

Compared to PIGD-PD patients, both the DRN and MRN exhibited reduced connectivity with the bilateral sensorimotor cortex, temporal cortex, occipital cortex, and left anterior cerebellum in TD-PD patients. Moreover, the DRN had hypoconnectivity with the left frontal cortex, bilateral parietal cortex, bilateral DCG, and posterior cerebellum, while the MRN showed hypoconnectivity with the right frontal cortex, bilateral occipital cortex, bilateral postcentral gyrus, right fusiform gyrus, and left insula (*p* < 0.05, FDR corrected; Figure 2, Supplementary Table S2).

### 3.3 Correlation analysis

**Figure 3:**
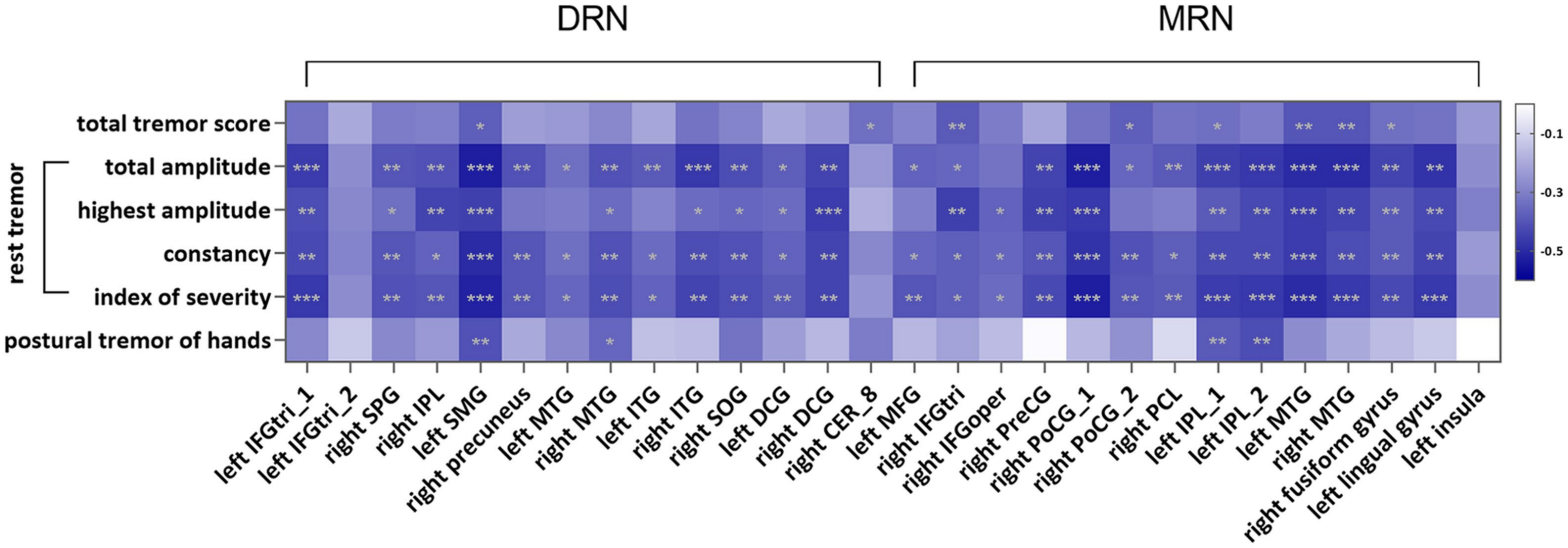
Heat map of correlation between connectivity of the raphe nuclei and tremor-related scores in the pooled PD group. The negative correlations are shown with blue, with darker shades representing stronger correlations. *: *p* < 0.05, **: *p* < 0.01, ***: *p* < 0.001, Bonferroni corrected. DRN, dorsal raphe nuclei; MRN, median raphe nuclei; CER_8, Cerebelum_8; DCG, median cingulate and paracingulate gyri; IFGoper, opercular part of inferior frontal gyrus; IFGtri, triangular part of inferior frontal gyrus; ITG, inferior temporal gyrus; IPL, inferior parietal, but supramarginal and angular gyri; MFG, middle frontal gyrus; MTG, middle temporal gyrus; PCL, paracentral lobule; PoCG, postcentral gyrus; PreCG, precentral gyrus; SMG, supramarginal gyrus; SOG, superior occipital gyrus; SPG, superior parietal gyrus.

In the pooled PD group, we found that FC of the raphe nuclei was negatively correlated with total tremor scores and all rest tremor-related features (Figure 3, Supplementary Table S3). The FC between the DRN and right middle temporal gyrus and right superior occipital gyrus, as well as the FC between MRN and left inferior parietal, supramarginal and angular gyri (IPL), were negatively associated with postural tremor score. No significant correlation was observed between the FC of the raphe nuclei and kinetic tremor score, PIGD score, rigidity scores, or bradykinesia scores.

In the TD-PD patients, we observed that FC between DRN and left DCG was negatively associated with highest rest tremor amplitude score; the FC between MRN and right postcentral gyrus showed a negative association with total rest tremor amplitude score; the FC between MRN and left IPL showed a negative correlation with postural tremor (Supplementary Table S4). No significant association was observed between the FC of the raphe nuclei and other tremor scores.

The FC between DRN and right precuneus showed a positive correlation with MoCA scores in the TD-PD group (Spearman *r* = 0.4512, *p* = 0.0175, Bonferroni-corrected). No significant association between the raphe nuclei FC and non-motor scores was observed in the pooled PD group. Additionally, no significant correlation was observed between FC of the raphe nuclei and SBR, neither in the pooled PD group nor in the TD-PD group.

## 4 Discussion

This study investigated alterations in the functional networks of the raphe nuclei in patients with TD-PD. Our findings demonstrate that FC of both the DRN and MRN is significantly reduced in TD-PD patients compared to HCs and PIGD-PD patients. Furthermore, FC of the raphe nuclei is significantly correlated with the severity of Parkinsonian tremor.

Compared to HCs, both TD-PD and PIGD-PD patients exhibited decreased connectivity of the DRN and MRN, consistent with a recent MRI study showing hypoconnectivity of DRN and MRN in PD patients (Wang et al., 2022). Previous postmortem and neuroimaging studies have revealed dysfunction of the serotonergic system in PD, including loss of serotonin neurons (Halliday et al., 1990; Buddhala et al., 2015), global deficiency of serotonergic markers (Commons, 2016), and reduced serotonin transporter binding (Doder et al., 2003; Politis et al., 2010; Qamhawi et al., 2015). Moreover, we found that both DRN and MRN exhibited more widespread hypoconnectivity in TD-PD compared to PIGD-PD, involving extensive cortical areas, basal ganglia, thalamus, limbic systems, and cerebellum, demonstrating that the raphe nuclei-related functional networks are more impaired in TD-PD than in PIGD-PD. Loane et al. reported that TD-PD patients showed a local reduction of serotonin transporter binding in the raphe nuclei, thalamus, and primary motor cortex (Loane et al., 2013). Previous studies also demonstrated a lower raphe serotonin transporter availability (Qamhawi et al., 2015) and peripheral platelet 5-HT level (Wang et al., 2023) in TD-PD compared to non-TD-PD patients. It is proposed that the basal ganglia and CTC circuits are critical for generating the onset of tremor and maintaining the tremor amplitude (Helmich, 2017). Local thalamic networks may generate tremor oscillations and subsequently propagate these oscillations to the cortex and basal ganglia (Helmich et al., 2012; Kim et al., 2017). Depletion of serotonergic projection is hypothesized to contribute to pathological activity in the basal ganglia and CTC circuits (Helmich and Dirkx, 2017). We observed hypoconnectivity between the raphe nuclei and multiple key nodes of the basal ganglia and CTC circuits (e.g., pallidum, thalamus, cerebellum, and motor cortex) in TD-PD patients, supporting the involvement of serotonergic dysfunction in the damage of basal ganglia and CTC circuits.

In TD-PD, compared to PIGD-PD, both DRN and MRN exhibited hypoconnectivity with extensive brain regions associated with Parkinsonian tremor, such as the middle frontal gyrus, IPL, middle temporal gyrus, and cerebellum (Mure et al., 2010; Benito-León et al., 2018; Li et al., 2022). Additionally, both DRN and MRN showed hypoconnectivity with the occipital cortex. Neural activity in the occipital lobe has been reported to be negatively correlated with total tremor score (Benito-León et al., 2018; Xiong et al., 2021). Furthermore, we found that the FC of both DRN and MRN was significantly negatively associated with tremor scores in PD, including total tremor score, postural tremor score, and rest tremor scores (constancy, amplitude, and index of severity). Our findings align with previous molecular imaging reports indicating that serotonergic function correlates with the severity of Parkinsonian tremor, providing support for the involvement of serotonergic dysfunction in Parkinsonian tremor (Politis et al., 2010; Loane et al., 2013; Qamhawi et al., 2015; Pasquini et al., 2018). Based on our results, it is likely that both DRN and MRN are involved in the pathogenesis of Parkinsonian tremor. Furthermore, similar to previous studies (Brooks et al., 1992; Pavese et al., 2006), the SBR showed no significant association with tremor scores. When controlling for the SBR as a covariate, the correlations between the raphe nuclei FC and tremor remained significant. These findings indicate that dopaminergic deficits did not significantly contribute to the observed correlations between the raphe nuclei FC and tremor severity, and that the latter is mainly linked to serotonergic impairments.

As the primary origin of central serotonin neurons, the DRN and MRN have distinct and complementary projections. The DRN innervation is more concentrated in the cerebral cortex and basal ganglion, whereas the MRN projections are more prominent in the frontal cortex, cingulate cortex, and hippocampal regions (Commons, 2016). The DRN and MNR also differ in physiological function (Calizo et al., 2011). For instance, neuronal activity in the DRN strongly correlates with tonic muscle tension (Trulson and Jacobs, 1979), while the MRN plays a critical role in regulating hippocampal theta oscillations and locomotion (Kusljic et al., 2003; Kocsis et al., 2006). Further study is needed to clarify whether the DRN and MRN have different roles in the pathogenesis of Parkinsonian tremor.

In addition, PIGD-PD patients exhibited reduced connectivity of DRN and MRN compared to HCs. A previous study found that serotonin levels in cerebrospinal fluid were significantly decreased and negatively correlated with the severity of akinesia and gait freezing in PIGD-PD patients (Iacono et al., 1997). In contrast, another study found that a 5-HT2 receptor antagonist could improve akinesia and gait in PD patients (Henderson et al., 1992). In the present study, there was no association between the FC of both DRN and MRN and the PIGD score in PD patients. The heterogeneity of these results suggests that the role of serotonergic dysfunction in PIGD requires further investigation. Meanwhile, the FC of the raphe nuclei-related networks is significantly less reduced in PIGD-PD patients than in TD-PD patients, suggesting that the serotonergic system is more involved in Parkinsonian tremor than in PIGD.

Some limitations exist in the present study. First, the sample size was relatively small, and large-scale studies are needed to verify our findings. Second, our patients did not record electromyography during fMRI scanning. Future studies should collect electromyography data to explore the relationship between the raphe nuclei-related network changes and tremor amplitude and frequency. Third, the location (brainstem) and small volume of DRN and MRN make them susceptible to motion and physiological noise (Beliveau et al., 2015). Even after applying strict criteria for head movement and using ICA to mitigate physiological noise, residual artifacts may still affect the results. Fourth, given the small size of the raphe nuclei and the inherent sensitivity of FC to ROI delineation parameters, alternative ROI definitions (e.g., different radii or anatomically shaped masks) will be used in future studies to assess the robustness of the results. Fifth, while trait sleepiness was controlled for using the ESS as a covariate, state sleepiness during the scanning session was not directly measured. Additionally, further longitudinal studies are warranted to explore the relationship between progressive raphe nuclei-related network alterations and the progression of Parkinsonian tremor.

## 5 Conclusion

In this study, we identified substantial hypoconnectivity in the raphe nuclei in patients with TD-PD. Moreover, our findings indicate that both DRN- and MRN-related functional networks exhibit significant correlations with the severity of Parkinsonian tremor in PD patients. These results suggest that both DRN and MRN may be involved in the pathogenesis of Parkinsonian tremor.

## Data Availability

The raw data supporting the conclusions of this article will be made available by the authors, without undue reservation.

## Footnotes

1
http://www.fmrib.ox.ac.uk/fsl

2
https://www.fil.ion.ucl.ac.uk/spm/software/spm12/

3
https://www.nitrc.org/projects/bnv/
