# Supplementary material for: Altered resting-state functional connectivity of raphe nucleus is associated with tremor in Parkinson’s disease
Source: Front Aging Neurosci. 2025 Dec 31;17:1709735. doi: 10.3389/fnagi.2025.1709735 (PMC12801054; doi:10.3389/fnagi.2025.1709735)
Supplement: Supplementary file 1 [file Data_Sheet_1.docx]

Supplementary Material

# Supplementary Table S1. Characteristics of the signal and noise components.

| **Features of**  **independent components** | **Signal** | **Noise** |
| --- | --- | --- |
| Spatial map | 1. relatively large clusters  2. localization in grey matter | 1. small and scattered clusters  2. located in the white matter, Cerebrospinal fluid, blood vessels, brain edges, and air-tissue interface |
| Time series | rhythmic, regular | rhythmless, irregular, fluctuate significantly |
| Power spectral density | low-frequency fluctuations (0.01~0.1Hz) | full wave spectrum oscillation with high-frequency spectrum dominance |

# Supplementary Table S2. Connectivity differences between TD-PD and PIGD-PD participants for dorsal and median raphe nuclei.

| ***cluster size*** | ***Voxel-level*** |  |  | ***MNI coordinates*** | | |
| --- | --- | --- | --- | --- | --- | --- |
|  | ***Brain region_Hemisphere*** | ***Peak  t-value*** |  | ***X*** | ***Y*** | ***Z*** |
| ***DRN, TD<PIGD*** | |  |  |  |  |  |
| **495** | **Supramarginal Gyrus_L** | **5.03** |  | **-52** | **-28** | **36** |
|  | Inferior parietal, but supramarginal and angular gyri_L | 4.64 |  | -45 | -45 | 61 |
|  | Middle occipital gyrus_L | 4.60 |  | -24 | -62 | 40 |
|  | Supplementary motor area_L | 4.50 |  | 0 | 0 | 72 |
|  | Precuneus_L | 4.36 |  | -14 | -48 | 68 |
|  | Superior occipital gyrus_L | 4.33 |  | -24 | -69 | 40 |
|  | Superior frontal gyrus, dorsolateral_L | 4.29 |  | -28 | 3 | 68 |
|  | Superior parietal gyrus_L | 4.09 |  | -21 | -65 | 47 |
|  | Paracentral lobule_L | 3.91 |  | -7 | -24 | 72 |
|  | Postcentral gyrus_L | 3.74 |  | -34 | -41 | 65 |
|  | Supramarginal gyrus_L | 3.43 |  | -62 | -31 | 29 |
| **28** | **Precuneus_R** | **4.91** |  | **7** | **-45** | **72** |
| **38** | **Middle temporal gyrus_R** | **4.67** |  | **52** | **-55** | **0** |
|  | Inferior temporal gyrus_R | 4.15 |  | 58 | -62 | -4 |
| **53** | **Superior occipital gyrus_R** | **4.64** |  | **24** | **-65** | **43** |
|  | Superior parietal gyrus_R | 4.16 |  | 17 | -62 | 58 |
|  | Precuneus_R | 3.64 |  | 7 | -62 | 61 |
|  | Middle occipital gyrus_R | 3.56 |  | 31 | -62 | 40 |
| **18** | **Median cingulate and paracingulate gyri_L** | **4.52** |  | **-7** | **-38** | **50** |
|  | Precuneus_L | 3.32 |  | -14 | -45 | 54 |
| **48** | **Inferior parietal, but supramarginal and angular gyri_R** | **4.39** |  | **45** | **-34** | **47** |
|  | Supramarginal Gyrus_R | 4.25 |  | 48 | -24 | 36 |
| **57** | **Inferior temporal gyrus_R** | **4.31** |  | **52** | **-55** | **-25** |
|  | Cerebelum_Crus1_R | 3.69 |  | 41 | -58 | -25 |
| **63** | **Inferior temporal gyrus_L** | **4.27** |  | **-52** | **-58** | **-22** |
|  | Cerebelum_6_L | 3.71 |  | -38 | -48 | -25 |
|  | Fusiform gyrus_L | 3.63 |  | -45 | -62 | -18 |
|  | Inferior temporal gyrus_L | 3.25 |  | -58 | -58 | -11 |
|  | Cerebelum_4_5_L | 3.11 |  | -28 | -34 | -25 |
| **72** | **Inferior frontal gyrus, triangular part_L_1** | **4.24** |  | **-41** | **34** | **14** |
|  | Middle frontal gyrus_L | 4.07 |  | -41 | 45 | 25 |
|  | Inferior frontal gyrus, triangular part_L | 3.89 |  | -48 | 34 | 22 |
| **22** | **Inferior frontal gyrus, triangular part_L_2** | **4.21** |  | **-45** | **31** | **4** |
|  | Temporal pole: superior temporal gyrus_L | 3.28 |  | -48 | 10 | -4 |
| **12** | **Cerebelum_8_R** | **3.97** |  | **21** | **-69** | **-47** |
| **18** | **Superior parietal gyrus_R** | **3.93** |  | **41** | **-48** | **65** |
|  | Inferior parietal, but supramarginal and angular gyri_R | 3.92 |  | 38 | -41 | 54 |
| **44** | **Median cingulate and paracingulate gyri_R** | **3.75** |  | **3** | **-7** | **32** |
|  | Median cingulate and paracingulate gyri_L | 3.71 |  | -3 | -3 | 36 |
|  | Superior frontal gyrus, medial_L | 3.51 |  | 0 | 17 | 43 |
| **14** | **Middle temporal gyrus_L** | **3.56** |  | **-52** | **-58** | **0** |
|  |  |  |  |  |  |  |
|  |  |  |  |  |  |  |
| ***cluster size*** | ***Voxel-level*** | |  | ***MNI coordinate(mm)*** | | |
|  | ***Brain region_Hemisphere*** | ***Peak  t-value*** |  | ***X*** | ***Y*** | ***Z*** |
| ***MRN, TD<PIGD*** | |  |  |  |  |  |
| **366** | **Middle temporal gyrus_R** | **5.45** |  | **52** | **-72** | **7** |
|  | Inferior temporal gyrus_R | 4.99 |  | 41 | -62 | -7 |
|  | Superior occipital gyrus_R | 4.97 |  | 24 | -89 | 29 |
|  | Inferior occipital gyrus_R | 4.48 |  | 48 | -76 | -7 |
|  | Middle occipital gyrus_R | 4.33 |  | 34 | -83 | 25 |
|  | Fusiform gyrus_R | 3.90 |  | 41 | -62 | -18 |
|  | Cuneus_R | 3.66 |  | 7 | -86 | 32 |
| **315** | **Middle temporal gyrus_L** | **5.18** |  | **-55** | **-69** | **4** |
|  | Middle occipital gyrus_L | 5.03 |  | -52 | -76 | 4 |
|  | Inferior occipital gyrus_L | 4.78 |  | -48 | -69 | -7 |
|  | Superior occipital gyrus_L | 4.27 |  | -17 | -93 | 25 |
|  | Fusiform gyrus_L | 4.08 |  | -38 | -65 | -11 |
|  | Cuneus_L | 3.58 |  | 0 | -89 | 18 |
| **93** | **Lingual gyrus_L** | **4.85** |  | **-10** | **-83** | **-11** |
|  | Cerebelum_4_5_L | 3.88 |  | -14 | -45 | -11 |
| **55** | **Middle frontal gyrus_L** | **4.81** |  | **-48** | **45** | **18** |
| **29** | **Inferior frontal gyrus, opercular part_R** | **4.69** |  | **48** | **7** | **22** |
|  | Rolandic operculum_R | 3.84 |  | 58 | 7 | 14 |
| **68** | **Precentral gyrus_R** | **4.69** |  | **41** | **-3** | **47** |
|  | Postcentral gyrus_R | 3.65 |  | 55 | -17 | 40 |
| **34** | **Inferior parietal, but supramarginal and angular gyri_L_1** | **4.62** |  | **-34** | **-48** | **47** |
| **14** | **Insula_L** | **4.45** |  | **-38** | **-7** | **4** |
|  | Rolandic operculum_L | 4.00 |  | -41 | -7 | 14 |
| **67** | **Fusiform gyrus_R** | **4.31** |  | **28** | **-62** | **-14** |
|  | Lingual gyrus_R | 4.02 |  | 17 | -62 | -7 |
|  | Calcarine fissure and surrounding cortex_R | 3.48 |  | 21 | -62 | 7 |
| **27** | **Postcentral gyrus_R_1** | **4.07** |  | **41** | **-38** | **65** |
|  | Inferior parietal, but supramarginal and angular gyri_R | 3.65 |  | 45 | -45 | 58 |
| **28** | **Inferior frontal gyrus, triangular part_R** | **3.98** |  | **41** | **38** | **7** |
|  | Middle frontal gyrus_R | 3.34 |  | 52 | 41 | 22 |
| **11** | **Postcentral gyrus_R_2** | **3.93** |  | **14** | **-48** | **72** |
| **16** | **Paracentral lobule_R** | **3.68** |  | **10** | **-31** | **79** |
| **10** | **Inferior parietal, but supramarginal and angular gyri_L_2** | **3.65** |  | **-41** | **-48** | **61** |

# Supplementary Table S3. Significant correlation between FC values for dorsal and median raphe nuclei and clinical variables in the pooled PD participants.

| **Clinical variables** | **Brain regions** | **r** | **p-value** |
| --- | --- | --- | --- |
|  |  |  | **(FDR corr.)** |
| ***DRN*** |  |  |  |
| total tremor score | Supramarginal gyrus_L | -0.38 | 0.0129 |
|  | Cerebelum_8_R | -0.33 | 0.0405 |
| postural tremor of hands | Supramarginal gyrus_L | -0.32 | 0.0041 |
|  | Middle temporal gyrus_R | -0.29 | 0.0242 |
| ***MRN*** |  |  |  |
| total tremor score | Inferior frontal gyrus, triangular part_R | -0.39 | 0.0088 |
|  | Postcentral gyrus_R _2 | -0.39 | 0.0120 |
|  | Inferior parietal, but supramarginal and angular gyri_L _1 | -0.36 | 0.0417 |
|  | Middle temporal gyrus_L | -0.40 | 0.0051 |
|  | Middle temporal gyrus_R | -0.40 | 0.0061 |
|  | Fusiform gyrus_R | -0.36 | 0.0400 |
| postural tremor of hands | Inferior parietal, but supramarginal and angular gyri_L _2 | -0.35 | 0.0083 |
|  | Middle temporal gyrus_L | -0.31 | 0.0026 |

| **Brain regions** | **total rest tremor amplitude** | |  | **highest rest tremor amplitude** | |  | **rest tremor constancy** | |  | **index of rest tremor severity** | |
| --- | --- | --- | --- | --- | --- | --- | --- | --- | --- | --- | --- |
|  | **r** | ***p*-value** |  | **r** | ***p*-value** |  | **r** | ***p*-value** |  | **r** | ***p*-value** |
|  |  | **(FDR corr.)** |  |  | **(FDR corr.)** |  |  | **(FDR corr.)** |  |  | **(FDR corr.)** |
| ***DRN*** |  |  |  |  |  |  |  |  |  |  |  |
| left IFGtri_1 | -0.24 | 0.0005 |  | -0.26 | 0.0029 |  | -0.28 | 0.0020 |  | -0.25 | 0.0004 |
| left IFGtri_2 | -0.45 | ns |  | -0.42 | ns |  | -0.42 | ns |  | -0.45 | ns |
| right SPG | -0.41 | 0.0068 |  | -0.37 | 0.0391 |  | -0.41 | 0.0070 |  | -0.43 | 0.0046 |
| right IPL | -0.37 | 0.0042 |  | -0.40 | 0.0016 |  | -0.35 | 0.0141 |  | -0.38 | 0.0053 |
| left SMG | -0.52 | <0.0001 |  | -0.48 | 0.0008 |  | -0.51 | 0.0001 |  | -0.53 | <0.0001 |
| right precuneus | -0.44 | 0.0045 |  | -0.38 | ns |  | -0.43 | 0.0061 |  | -0.43 | 0.0054 |
| left MTG | -0.35 | 0.0401 |  | -0.34 | ns |  | -0.34 | 0.0405 |  | -0.36 | 0.0219 |
| right MTG | -0.40 | 0.0043 |  | -0.36 | 0.0321 |  | -0.40 | 0.0048 |  | -0.42 | 0.0032 |
| left ITG | -0.38 | 0.0091 |  | -0.31 | ns |  | -0.37 | 0.0274 |  | -0.37 | 0.0177 |
| right ITG | -0.43 | 0.0004 |  | -0.35 | 0.0273 |  | -0.43 | 0.0024 |  | -0.43 | 0.0011 |
| right SOG | -0.42 | 0.0026 |  | -0.38 | 0.0244 |  | -0.43 | 0.0035 |  | -0.43 | 0.0031 |
| left DCG | -0.38 | 0.0113 |  | -0.38 | 0.0353 |  | -0.38 | 0.0151 |  | -0.39 | 0.0081 |
| right DCG | -0.37 | 0.0016 |  | -0.41 | 0.0007 |  | -0.40 | 0.0014 |  | -0.38 | 0.0013 |
| right CER_8 | -0.23 | ns |  | -0.20 | ns |  | -0.28 | ns |  | -0.25 | ns |
| ***MRN*** |  |  |  |  |  |  |  |  |  |  |  |
| left MFG | -0.37 | 0.0146 |  | -0.33 | ns |  | -0.37 | 0.0248 |  | -0.40 | 0.0067 |
| right IFGtri | -0.38 | 0.0228 |  | -0.47 | 0.0010 |  | -0.41 | 0.0112 |  | -0.40 | 0.0131 |
| right IFGoper | -0.38 | ns |  | -0.42 | 0.0175 |  | -0.39 | 0.0226 |  | -0.39 | 0.0276 |
| right PreCG | -0.43 | 0.0012 |  | -0.45 | 0.0012 |  | -0.42 | 0.0048 |  | -0.44 | 0.0024 |
| right PoCG_1 | -0.53 | <0.0001 |  | -0.49 | <0.0001 |  | -0.50 | <0.0001 |  | -0.54 | <0.0001 |
| right PoCG_2 | -0.37 | 0.0234 |  | -0.35 | ns |  | -0.42 | 0.0045 |  | -0.41 | 0.0052 |
| right PCL | -0.40 | 0.0085 |  | -0.34 | ns |  | -0.40 | 0.0138 |  | -0.41 | 0.0093 |
| left IPL_1 | -0.45 | 0.0009 |  | -0.42 | 0.0094 |  | -0.47 | 0.0018 |  | -0.48 | 0.0007 |
| left IPL_2 | -0.50 | 0.0003 |  | -0.47 | 0.0023 |  | -0.46 | 0.0020 |  | -0.50 | 0.0004 |
| left MTG | -0.49 | 0.0001 |  | -0.47 | 0.0005 |  | -0.48 | 0.0006 |  | -0.50 | 0.0001 |
| right MTG | -0.49 | 0.0001 |  | -0.45 | 0.0011 |  | -0.45 | 0.0025 |  | -0.49 | 0.0003 |
| right fusiform gyrus | -0.44 | 0.0011 |  | -0.40 | 0.0084 |  | -0.41 | 0.0087 |  | -0.44 | 0.0020 |
| left lingual gyrus | -0.47 | 0.0002 |  | -0.42 | 0.0020 |  | -0.45 | 0.0012 |  | -0.47 | 0.0003 |
| left insula | -0.28 | ns |  | -0.31 | ns |  | -0.24 | ns |  | -0.28 | 0.0279 |

***Abbreviation:*** DRN, dorsal raphe nuclei; MRN, median raphe nuclei; DCG, median cingulate and paracingulate gyri; IFGoper, opercular part of inferior frontal gyrus; IFGtri, triangular part of inferior frontal gyrus; ITG, inferior temporal gyrus; IPL, inferior parietal, but supramarginal and angular gyri; MFG, middle frontal gyrus; MTG, middle temporal gyrus; PCL, paracentral lobule; PoCG, postcentral gyrus; PreCG, precentral gyrus; SMG, supramarginal gyrus; SOG, superior occipital gyrus; SPG, superior parietal gyrus.

# Supplementary Table S4. Significant correlation between FC values for dorsal and median raphe nuclei and clinical variables in the TD-PD participants.

| **Clinical variables** | **Brain regions** | **r** | **p-value (FDR corr.)** |
| --- | --- | --- | --- |
| ***DRN*** |  |  |  |
| Highest rest tremor amplitude | Median cingulate and paracingulate gyri_L | -0.48 | 0.0424 |

| ***MRN*** |  |  |  |
| --- | --- | --- | --- |
| Total rest tremor amplitude | Postcentral gyrus_R | -0.49 | 0.0406 |
| Postural tremor of hands | Inferior parietal, but supramarginal and angular gyri_L_1 | -0.53 | 0.0144 |
|  | Inferior parietal, but supramarginal and angular gyri_L_2 | -0.56 | 0.0069 |


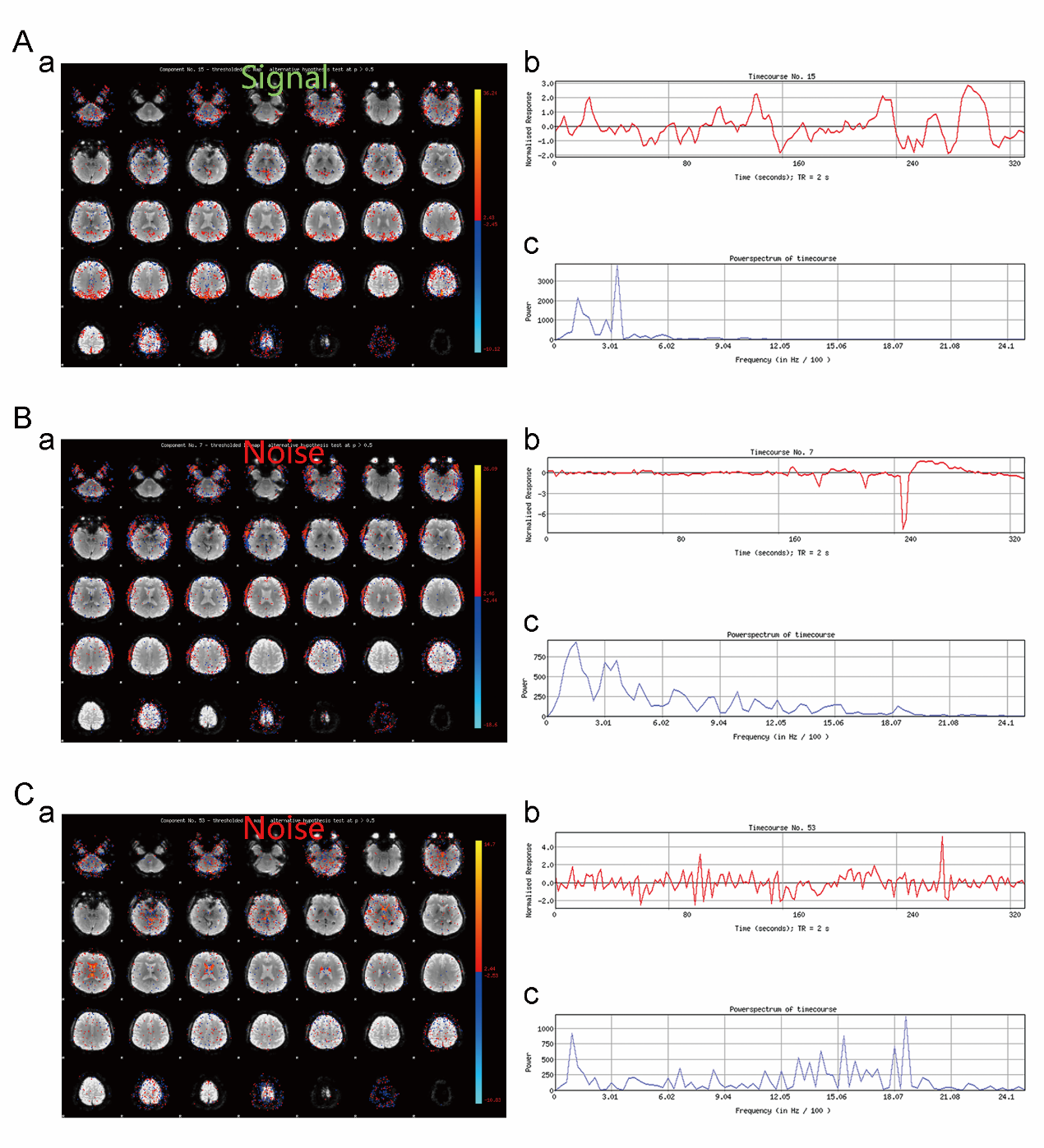


# Supplementary Figure S1. Examples of signal (A) and noise (B-C) components. a, The spatial map of clusters. b, The time series. c, The power spectral density.


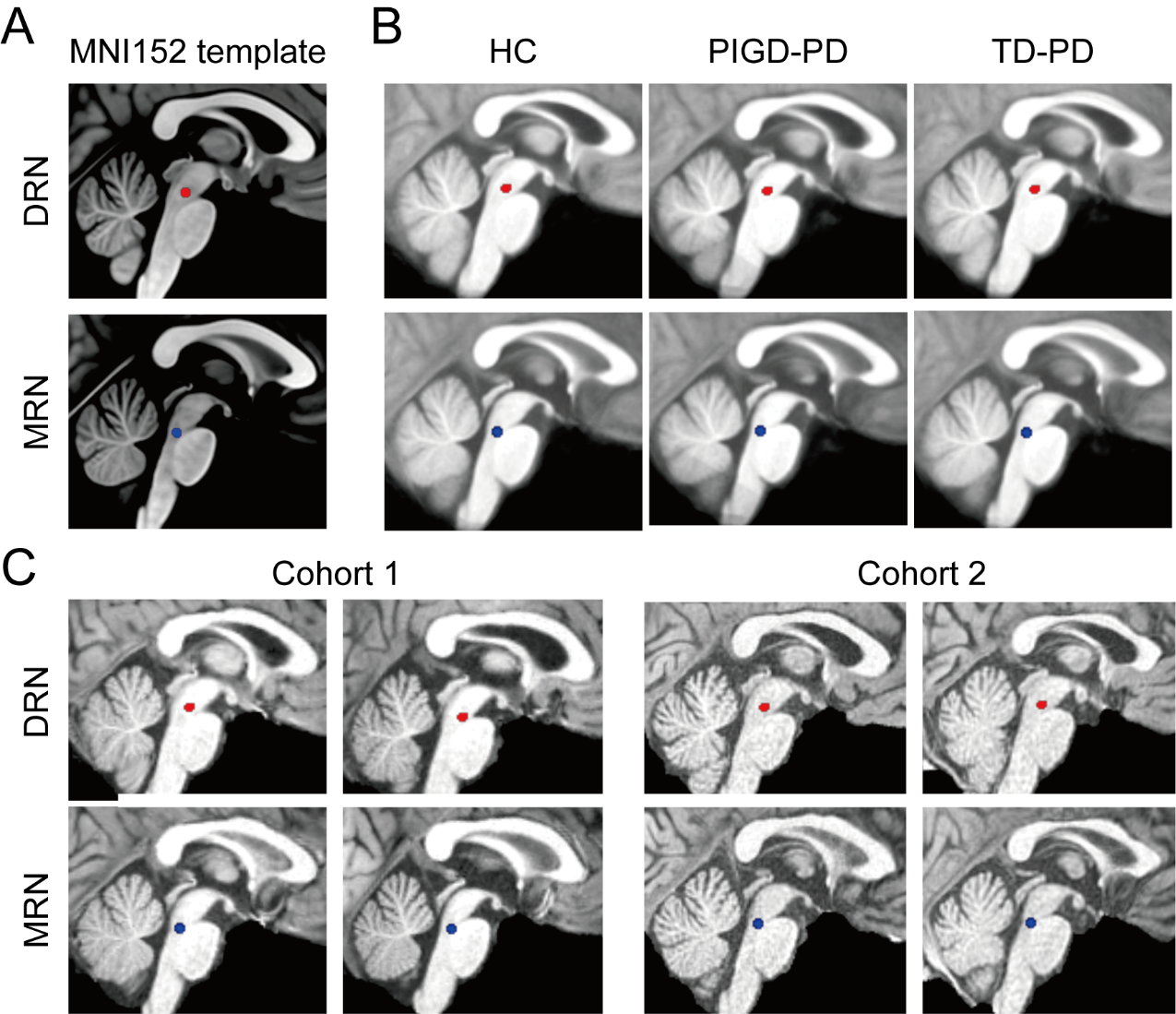


# Supplementary Figure S2. Position of the two raphe-ROI seeds. Sagittal views of the seeds are shown on (A) the MNI152 template, (B) the group-averaged T1-weighted images, and (C) representative individual’s T1-weighted images.


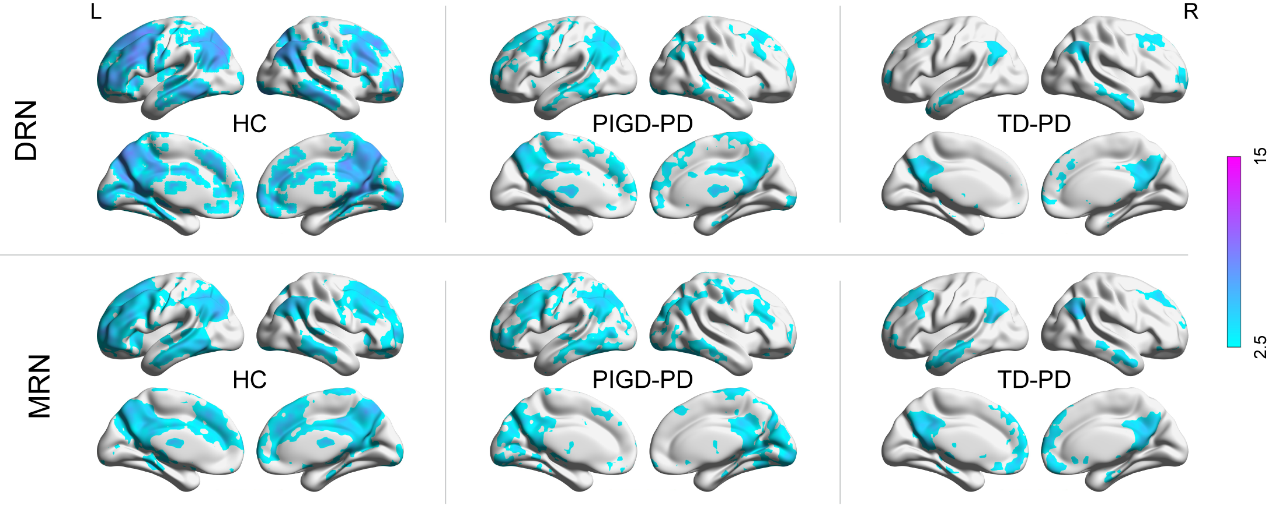


# Supplementary Figure S3. Within-group functional connectivity maps of raphe nuclei in TD-PD, PIGD-PD groups, and HCs. Results are thresholded at FDR-corrected voxel-level p < 0.05 (cluster size ≥10 voxels). DRN, dorsal raphe nucleus; MRN, median raphe nucleus; HC, healthy control; PIGD-PD, postural instability and gait difficulty‐dominant Parkinson’s disease; TD-PD, Tremor-dominant Parkinson’s disease.


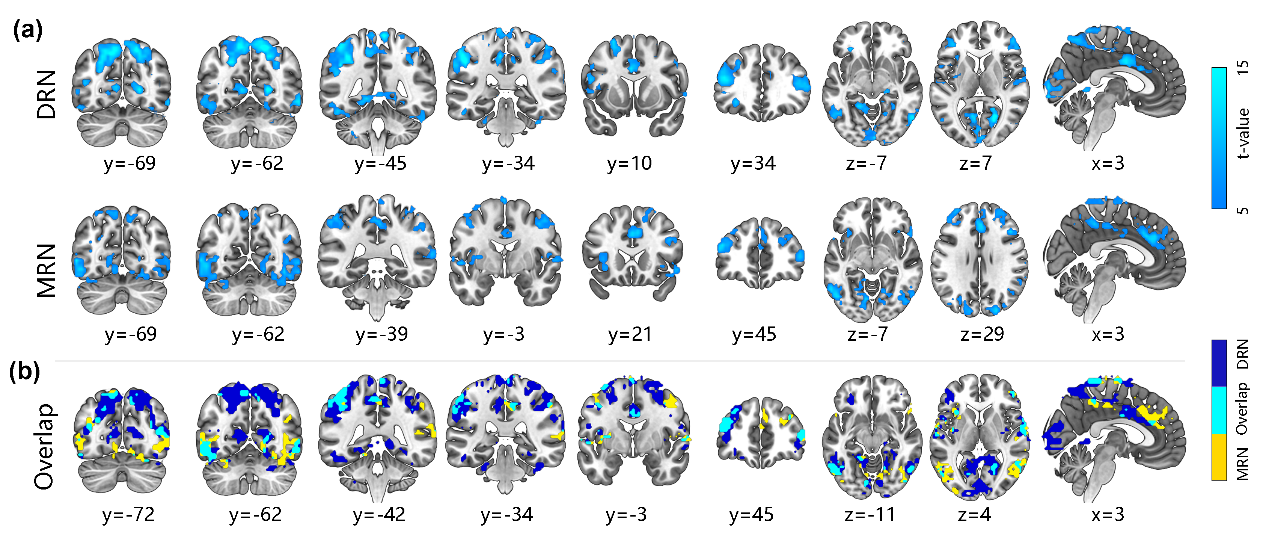


# Supplementary Figure S4. Differences of functional connectivity with the raphe nuclei among TD-PD, PIGD-PD groups, and HCs.

# (a) Significant group differences in FC values for the DRN and MRN (p-value < 0.05, FDR-corrected, MNI152 space, cluster size ≥10 voxels). (b) Similarities and differences in spatial maps of FC between DRN and MRN. The brain areas with orange are unique to DRN, those with blue are specific to MRN, and those with cyan are common to both.
